# Partnering with Young Parents to Improve Early Hearing Detection and Intervention Programmes

**DOI:** 10.3390/children12050629

**Published:** 2025-05-13

**Authors:** Genevieve Choi, Holly Teagle, Suzanne C. Purdy, Andrew Wood

**Affiliations:** 1 Department of Audiology, The University of Auckland, Auckland 1023, New Zealand; holly.teagle@auckland.ac.nz; 2 School of Psychology, The University of Auckland, Auckland 1023, New Zealand; sc.purdy@auckland.ac.nz; 3 Department of Surgery, The University of Auckland, Auckland 1023, New Zealand; andrew.wood@auckland.ac.nz

**Keywords:** adolescent parents, early hearing detection and intervention, engagement, loss to follow-up

## Abstract

**Background:** Early Hearing Detection and Intervention (EHDI) programmes must partner effectively with families navigating complex circumstances. Adolescent parents (APs) in Teen Parent Units (TPUs) represent a dynamic group demonstrating resilience as they balance childcare, education, and their own developmental journeys. This study explores their understanding of infant hearing, sources of knowledge, and the development of an effective teaching tool. **Methods:** A qualitative study was conducted with AP learners at a TPU in Aotearoa, New Zealand. Following a period of relationship-building, three focus groups were held. Data were analysed using content analysis and reflexive thematic analysis. **Results:** AP learners demonstrated a strong awareness of multisensory interactions. Major sources of knowledge included their relational interactions with people they trusted (midwives and family members), rather than social media. Four key themes emerged in the teaching tool’s development: (1) the effectiveness of multimodal teaching tools, (2) the benefits of peer-supported group learning, (3) the impact of high strain, and (4) the importance of Te Ao Māori (a Māori worldview). **Conclusions:** This study highlights the importance of culturally grounded health interventions for families navigating complex life circumstances. Group-based learning fostered peer support, hands-on multimodal teaching was effective, and culturally relevant materials and pedagogies enhanced engagement. EHDI programs may more effectively support infants from families navigating complex circumstances by collaborating with trusted support people, integrating with wraparound care networks, utilising safe and familiar settings, and delivering interventions in an engaging and culturally appropriate manner.

## 1. Introduction

Early Hearing Detection and Intervention (EHDI) programmes are widespread internationally [1] due to the importance of identifying and addressing hearing loss early in life. Early intervention capitalises on the critical window of neuroplasticity for auditory and language development [2].

Early-life brain development is influenced by the stimulation of all the primary senses, as well as complex multisensory experiences [3]. When an infant does not have access to the language used by their main carers, it places an additional burden on their developing brain, and this is the case for infants with hearing loss, born to parents and main carers who use spoken language [4]. Numerous factors influence brain development, and the cumulative effects of multiple adverse conditions can be observed in measures of a child’s executive function (EF) as early as the preschool years [5]. Examples of broader systemic factors influencing development include housing instability, inequitable access to education, adolescent parenthood, financial hardship, and parenting without consistent support networks [5,6]. These systemic factors can create significant challenges for families, affecting their access to and engagement with EHDI services. To support optimal hearing and language development, EHDI programmes must meaningfully partner with families navigating complex circumstances that may increase the demands of raising a child with hearing loss.

Equity in health outcomes is a central consideration for EHDI programmes, as disadvantage is often intergenerational and disproportionately affects marginalised populations [6,7]. While high screening coverage rates have been reported in the literature, there is increasing emphasis on reducing loss to follow-up/documentation (LTFU) rates, which disproportionately affect families under high strain [7,8,9,10]. In New Zealand, families facing material hardship experience delays in diagnostic assessments and poorer screening coverage, mirroring international trends [11,12].

This study focuses on a unique cohort of adolescent parent (AP) learners, who are completing high school qualifications at a Teen Parent Unit (TPU). These young parents strive to balance raising infants and completing high school qualifications, as well as navigating their own adolescent development. This triad of responsibilities reflects the complexity facing and resilience required of these young parents. In addition, adolescent parenthood often occurs alongside systemic challenges such as housing instability, limited access to resources, and disrupted family support networks [13,14]. In this paper, the cumulative challenges described by Wallander [5] are referred to as “strain”, recognising their potential to influence developmental trajectories. The language environment of the home is a key factor in infants’ cognitive and language development [15,16]. A crucial aspect of any EHDI programme is supporting main carers to provide a language-rich environment for their infant [17,18]. Variations in home language environments are influenced by factors such as unequal access to education [19,20], economic hardship, caregiver mental health challenges [21,22], and other intersecting systemic conditions [23]. Another consideration is that AP learners themselves may have had fewer opportunities to access language-enriching environments compared to their age-matched, non-parent peers [24,25]. This population represents an important group for EHDI to engage with, both because they are navigating complex circumstances and because they may benefit from additional support in fostering a language-rich home environment.

Adolescent parents can experience systemic inequities and stigma [26,27,28]. This study takes a strengths-based approach [29], acknowledging the broader structural factors that shape the experiences of adolescent parents [14,30]. While adolescent parenting has historically been associated with risk-taking behaviours, emerging evidence supports the theory of preselection [13,31], emphasising the role of early-life exposures such as economic hardship, racism, and childhood trauma [14,32]. Understanding and addressing these intersecting challenges is critical to supporting families and improving outcomes for their children.

Research on interventions with families navigating multiple challenges highlights the importance of targeted engagement strategies. Randomised controlled trials have demonstrated the benefits of such interventions, but many existing studies in this area are limited in their ability to recruit and maintain engagement with the intended population [33,34]. Families navigating systemic barriers may face multiple challenges in accessing health services and participating in research studies [33,35]. The TPU provides a unique setting to explore these issues, as it offers wraparound care, including transportation, childcare, and access to nurses, social workers, and psychologists.

This study builds on this foundation of support and an investment of time in building trust and becoming familiar with the TPU staff, systems, culture, and students before formal data collection was undertaken. By working closely with AP learners, this research aims to inform strategies for improving engagement with families navigating complex circumstances who are identified by the EHDI programme.

This study addresses three key questions:

1. Understanding: What do adolescent parents in high-strain families know about hearing in infants under the age of two?
2. Sources of Knowledge: What sources of information have shaped their understanding?
3. Teaching Tool Development: What does a teaching tool need to contain to effectively communicate the importance of early hearing to families experiencing high strain?

By gathering insights from a group of AP learners in a semi-structured and safe environment, this study seeks to identify strategies to improve engagement with families experiencing high strain, which could reduce LTFU rates within the EHDI programme and enhance equity in early-childhood hearing and language outcomes.

## 2. Materials and Methods

### 2.1. Design

This qualitative study involved a period of whakawhanaungatanga (rapport building), followed by three focus group sessions (FG1, FG2, and FG3). The focus group sessions were audio recorded, allowing transcription and analysis. The overarching philosophy of this study is influenced by Kaupapa Māori (KPM) Research [36], as well as codesign methodology [37].

This study primarily aligns with a constructivist knowledge creation, emphasising the co-construction of knowledge through participant interaction and context [38]. It is influenced by Indigenous knowledge systems, namely KPM research. It seeks to generate practical and meaningful insights. It includes small amounts of quantitative methods, which are used to support the qualitative themes identified or to help determine the appropriate descriptive techniques to use when the volume of data was low.

### 2.2. Positionality

The research team included a PhD candidate (GC), and her three PhD supervisors (HT, AW, and SCP). GC has a background in clinical paediatric audiology in the health sector. HT is an associate professor with a background in paediatric audiology and a clinical director for a paediatric cochlear implant centre. AW is a senior lecturer who is also a practicing ear, nose, and throat surgeon. SCP is a professor of psychology with a clinical audiology background and an extensive research background, including qualitative research methods and KPM methodology.

All the researchers are parents themselves; however, none has previous, close experience working with adolescent parents. GC is Pākehā (NZ European) and spent her primary school years in a Māori-led bilingual unit within a mainstream school. Amongst the wider research team, SCP is Māori (Te Rarawa, Ngāi Takoto), and AW and HT are Pākehā.

### 2.3. Setting

The location for the interactions with the AP learners’ group was at their classroom site, which is adjacent to a mainstream high school. This site includes a co-located daycare for their children and rooms used routinely by their employed social worker, nurse, and psychologist and on occasion by Well-Child providers. It also included space that the lead teacher offered for use by the research team, as the whole unit was described as the learners’ safe space.

### 2.4. Study Timeline

GC had three face-to-face meetings with the lead teacher onsite at the classroom prior to ethics approval and the first whakawhanaungatanga session, from October 2022 through to May 2024. Further details of the study timeline are shown in Table 1. 

During Phase 1, GC arranged a mid-morning snack for the class to share and gave a short presentation about herself, her role as a hospital-based paediatric audiologist, and her current study. A research flyer was given out (see Supplementary Material S1). Over the six weeks of rapport building, GC was in class for one half-day a week. As she worked alongside the AP learners, she was available for questions and chats about health. As well as chats, during this rapport-building time, GC was available for conversations, help with health navigation, and for supporting familiarisation with hearing measurement methods.

The lead teacher identified AP learners who met the entry criteria and provided them with the participant information sheet (see Supplementary Material S1) and consent forms. From the class of 30, 7 volunteered and were available to participate on the day of FG1. Each session was less than one hour. There was three weeks between FG1 and FG2 and one week between FG 2 and FG3. The study occurred over term one and two, to avoid overlap with the end of school year exams.

### 2.5. Participant Eligibility Criteria

#### 2.5.1. Inclusion Criteria

To be included in the study, participants needed to provide consent, be enrolled in the local Teen Parent Unit class, speak English, and meet either of these criteria:

- Being a first-time parent of an infant who is under 2 years of age;
- Being hapū (pregnant) with their first infant.

#### 2.5.2. Exclusion Criteria

Learners were ineligible to participate in the study if any of the following applied:

- They were unable to provide consent (due to their own diminished understanding or comprehension);
- They had children older than 2 years of age;
- They did not attend FG2 (in reference to participating in FG3).

### 2.6. Participants

To protect participants’ anonymity, pseudonyms have been used (see Table 2). All the participants self-identified as Māori and female. The ages of the participants ranged from 14 to 21 years.

There is one portion of the results section in which the participants’ pseudonyms are not used, and instead the A/B comparison uses these terms. This is to further help maintain anonymity.

### 2.7. Data Collection

The three FGs were under 1 h in length and were audio recorded. The recordings were then transcribed. The transcription included reflexive memos written by GC and explanatory notes. The explanatory notes included information about the intention of the message from the participant at that point in the discussion. The understanding of the intention of the participant was influenced by their tone of voice (captured by the audio recording), and their body language (from the researcher’s observation), and the surrounding conversation and interactions with peers (elucidating the meaning of colloquial slang). The explanatory notes helped to capture the participants’ intended meaning in the written form.

FG1 involved four prompts, which were provided verbally and with a visual printout of the prompt (Supplementary Material S2). FG2 was predominantly a teaching session, and the teaching tools used are shown in Supplementary Material S2.

Personal reflexivity [39], guided GC in journaling information (observations, reflections, and realisations) over the 19 months as she became familiar with the class, teachers, and how the system operated. Discussion between GC and her three supervisors took place as a group and with smaller subsets, facilitating further reflections, which were then journalled.

### 2.8. Data Analysis

GC became familiar with the data through the transcribing process, which included listening to the audio recordings many times to ensure correct capture of the tone and intended meaning of the participants.

To address aim 1 of this research, “Understanding” content analysis was used to analyse the FG1 transcript in three ways [40].

Firstly, the raw transcript was broken into data segments. These segments were categorised as being closely related to infant hearing or peripherally related. The closely related segments were further categorised into the three researcher-described categories: sounds infants hear, hearing checks, and infant development. The corresponding verbatim section of transcript is displayed and was edited minimally to improve readability (sections removed indicated with ellipses and sections added indicated by square brackets).

Next, the prompted topics that generated an absence of discussion were reported. It is acknowledged that valuable insights can be gained using this technique [41]. It contributes towards avoiding the biases that occur if only the content that prompted discussion is analysed.

The final method employed was a measurement of the time spent on each topic [41] and labelling whether the topic was prompted by the researcher or led to by the participants. The time spent on topics closely related to infant hearing included the time used by the researcher for the prompts.

To address aim 2, “Sources of Knowledge”, reflexive thematic analysis (RTA) was performed on the entire FG1 transcript [42]. Including the discussion of sources of knowledge for all health topics, rather than only those that related to hearing, provided a larger data set and a more meaningful analysis. This thematic analysis used inductive coding. An iterative process mapped the relationship between codes and grouped them into themes. After several rounds, the final themes were identified. This process acted to refine the codes, as the final set of themes excluded some codes.

A small portion of FG2 was relevant to one of the themes identified in the thematic analysis of FG1. It was not prompted by the researcher and was initiated by the participants. It has therefore been incorporated into the thematic analysis of FG1 and is labelled as originating from FG2 in the results section.

**Figure 1:**
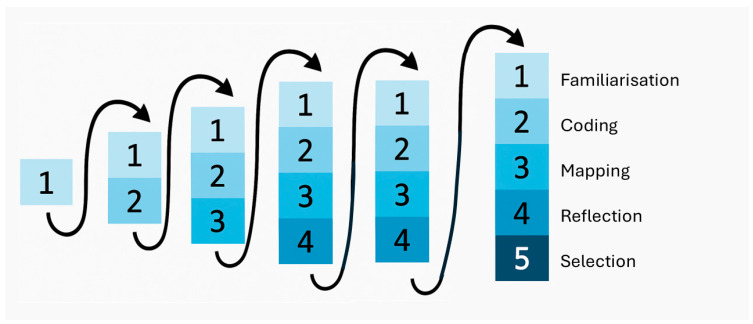
A visual representation of reflexive thematic analysis (RTA), illustrating the spiral rounds of iterative interaction with the raw data, the inclusion of further processing on each round of interaction, and the emphasis on reflection. The numbers in the figure represent the following steps: (1) Interaction with the raw data (transcripts). In the initial cycle: familiarisation (transcription, re-listening to audio, writing explanatory notes, and writing memos). In subsequent cycles: re-familiarisation (reading original transcripts, memos, and journaled reflections). (2) Coding (open and inductive) using NVivo. (3) Grouping and mapping codes into themes. (4) Reflection, considering the strength-based approach prescribed by Kaupapa Māori research; learnings from broader interactions over 19 months (with the whole class, teachers, and the learning system); and the original aims of the study. (5) Selection of the final set of themes and supporting examples (either verbatim quotes or quantitative measures derived from the audio recordings).

FG2 and FG3 were analysed using RTA, as it supports an iterative interaction with the transcripts while also embracing the researchers’ reflections [43]. These reflections were shaped by over 19 months of interaction between GC and the lead teacher, the class of AP learners, the three other teachers, the overall operation of the learning environment, and the discussion of these interactions with the wider research team. As well as RTA enabling integration of the themes identified from the transcripts with reflections on the broader interactions with the class, this technique also facilitated mindfulness of the strengths-based approach prescribed by KPM research [36,44] and the original aims of the study (see Figure 1).

### 2.9. Reciprocation

A mid-morning snack was provided at several of the rapport-building sessions for the learners to share. The learners who attended the focus group sessions received a NZ$25 gift card at each session. During the whakawhanaungatanga time, questions and discussions about hearing loss and general health were welcomed. Hearing checks for infants were offered onsite at the request of any AP learners.

## 3. Results

### 3.1. Understanding

The total content of FG1 that related to infant hearing is shown in Table 3. This includes conversations prompted by the researcher, as well as those led by the participants. The content has been summarised as belonging to three categories: sounds infants hear, hearing checks, and infant development (which includes the first 1000 days, infant communication, and infant emotions). As this is a content analysis and includes the entire content (of FG1, which related to infant hearing), in contrast to the RTA that has been used in the rest of this study, the supporting evidence is not described as quotes and is instead labelled *transcript excerpts*.

The participants in FG1 showed awareness that the stimulation of the senses is important for their infants’ early-life development. The majority of their discussion was about infant development, including infant/main carer communication and the emotional aspect of this interaction. This may reflect greater knowledge of this topic or interest in this topic compared to the narrower topics of infant’s hearing and hearing checks. Communication between infants and their main carers, including the emotional aspects, are a complex and multisensory experience for the infant. AP learners showed knowledge of the importance of these interactions.

Experiences with newborn hearing screening varied. While most participants found the screening uneventful and convenient, two participants felt uneasy about the machine touching their infant’s soft spot.

Participants recognised the importance of early interactions between infants and their main carers, noting that engagement is more meaningful than passive sensory experiences like listening to music. They were aware of how pre-verbal infants communicate and comprehend familiarity. There was some suggestion that the AP learners may be more aware of other senses than hearing alone in these multisensory experiences, e.g., “absorb what they see and feel” and “when they see Mum, they are happy” [following a conversation about the familiarity of sounds]. In the final moments of FG1, however, an AP learner was able to describe behaviours of her infant that related to their hearing sense alone and related to language-relevant hearing over their first 12 months of life (Table 3, row 12). This suggests the topic of infant hearing may have initially been abstract or novel for many AP learners; however, for at least one AP, by the end of FG1 they were able to articulate an understanding of infant hearing as it relates to communication and language development. As she was able to describe the subtle signs that relate to this developmental milestone, we can infer that they are well bonded, and she is attuned to her infant’s needs. It is noteworthy that although study aim 1 was to assess knowledge of AP, there is a complex interaction between knowledge and other aspects of main carer/infant relationships.

There was an absence of discussion following some of the researcher’s prompts regarding infant hearing and development. These are described in Table 4. The absence of discussion could be due to several reasons, including an absence of knowledge, individual participants not wanting to voice their answer due to an adolescent style of communication with the adult researcher, or individual participants not wanting to voice their answer due to a peer group dynamic. In relation to the prompt the “first 1000 days”, one participant verbalised an accurate understanding of the concept, “*Isn’t that the first three years are crucial*?” (Brooke). The conversation of the wider group of participants flowed quickly onto a different topic before Brooke’s thoughts about this could be further explored. Even with further prompts later in the focus group, GC was unable to prompt Brooke to share any further thoughts about this topic.

**Figure 2:**
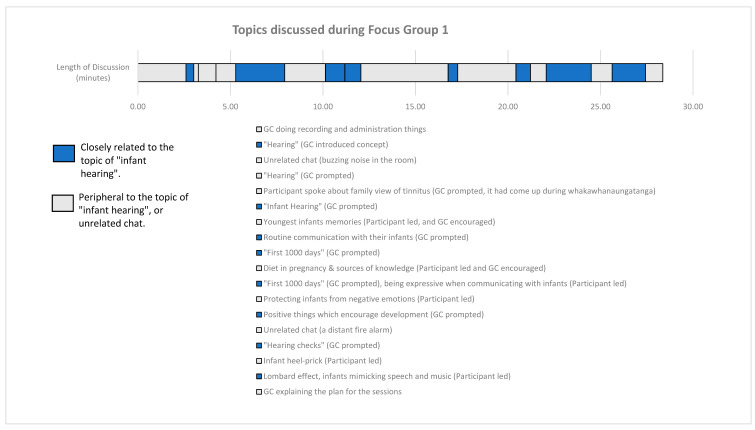
Topics discussed during FG1, which includes the time spent on each topic, and whether the topics were prompted by GC, or flowed onto by the natural conversation of the participants. Topics with quotation marks are the formal prompts that had a supporting visual prompt.

Figure 2 shows the time spent on each topic and whether the topic was prompted by the researcher or led to by the participants. The total time spent on FG1 was short (around 28 min). There were longer blocks of time spent discussing peripheral topics compared to the topics closely related to infant hearing. The time spent on infant hearing-related topics included the time used by the researcher for the prompts, meaning this difference in time of the participants’ discussion was more than what is visually displayed in Figure 2. Participant-led topics included diet during pregnancy, protecting infants from negative emotions, and the newborn heel prick.

There was evidence within FG2 and FG3 of the participants trying hard to answer the researcher’s questions as best they could. This implies that the comparatively short time spent on discussing infant hearing may be an indicator that AP learners had little to say on the topic of infant hearing. This may suggest that opportunities to learn about infant hearing have been limited for AP learners.

### 3.2. Sources of Knowledge

A thematic analysis of FG1 (both researcher-prompted and participant-led topics) resulted in three main themes. The first two were identified through inductive coding and the third through deductive coding, considering aim 2 of this study. The identified themes were: interactions with the health sector, communication with infants and sources of knowledge.

#### 3.2.1. Interactions with the Health Sector

The AP learners’ interactions with the health sector was a reoccurring theme throughout the discussion, including public health messages, health services, and health literacy. There was evidence of a high level of health literacy for some topics. This suggests that there has likely been a previous positive direct or indirect interaction with a public health message. For example, learners knew that the infant in utero had developed their hearing organs by 20 weeks’ gestation, that the first few years of life were crucial for infant development, and learners were knowledgeable about techniques to support their infants’ growth and development.

*I reckon kids interact more when you interact with them. Doing whatever it may be… (Erin, FG1)*

As well as knowledge, there was evidence of finding some public health messages overly restrictive and cumbersome.

*…like what are we supposed to eat during pregnancy? [said in a defensive manner] (Abby, FG1)*

This emotive response to public health messages came from the new awareness by the AP learners that the “first 1000 days” concept included pregnancy. Although there was knowledge that the first few years of life are crucial for development, the group of learners did not appear to be aware that this crucial time for development included the time in utero. There was evidence of the AP learners not following food safety recommendations during pregnancy.

*So, I ate BK [fast food] chickens, and drank Coca-Cola...most of the time. (Abby, FG1)*

*I drunk coffee... (Clara, FG1)*

*Yeah same, I ate seafood, I ate everything. (Brooke, FG1)*

*Same, I didn’t care. Well, it’s not that I didn’t care, my body was just… (Clara, FG1)*

*Wanting it, aye? (Erin, FG1)*

*Yeah. (Clara, FG1)*

*I ate everything you weren’t allowed. “Just stay away from raw sea food” or whatever, I still ate it. (Erin, FG1)*

The discussions showed that some AP learners made decisions that did not align with public health advice regarding food safety during pregnancy. During the discussions, there was no evidence that the learners had knowledge about why food safety guidelines for pregnancy may exist, suggesting that health literacy may be a driver behind these choices. Not following the recommended public health advice occurred for other health topics also, for example not partaking in prenatal health care. The drivers behind this are likely more complex than health literacy.

*…I didn’t tell anyone. Only one person. (Clara, FG1)*

*So not seeing a midwife was part of keeping it a secret? (Researcher, FG1)*

*[head nod showing agreement] And my sister actually, two people. (Clara, FG1)*

*Was your sister younger or older? (Researcher, FG1)*

*She’s older. (Clara, FG1)*

*She didn’t encourage you to have a scan or anything? Do the check-ups? (Researcher, FG1)*

*No. She’s only like a year older than me. (Clara, FG1)*

There appeared to be an absence of knowledge amongst the learners about the relationship between population health research and the evidence-based public health messages they were receiving. This relationship includes complex health literacy topics, such as individual risk, the predictive value gained from population health data, and survivor bias. Their lack of health literacy in this area may be typical of a group of learners completing high school-level qualifications.

*‘Cause in the olden days, they didn’t know all of that [information about diet restrictions], and their babies still came out fine. (Brooke, FG1)*

*Honest, just look at all of us! Nothing wrong with us! [said in an assertive manner] (Clara, FG1)*

*Sometimes what they say doesn’t really affect you. [said following reports of eating seafood and feeling good afterwards] (Erin, FG1)*

#### 3.2.2. Communication with Infants

There was evidence from the discussions that the learners were aware of the importance of interacting with their infants and were aware of methods that supported infant development. The discussions included many infant senses (hearing, sight, touch), as well as the emotional aspect of the interactions between themselves and their infants.

*Music is a good tool for like happiness with babies I reckon. Cause you play an Old McDonalds song, and you just see the baby’s head just nodding, nodding, and you’re like what!? [said with an expression of awe] (Erin)*

*…He likes mimicking people and their voice, and he likes to sing songs, like, he doesn’t sing it proper but…you know… (Brooke, FG1)*

*Can you tell the tune? (Researcher, FG1)*

*Yeah. (Brooke, FG1)*

There was a natural progression of the conversation towards protecting infants from negative influences: however, with prompting, there was equally as much knowledge about positive interactions with infants that encourage development.

*I got told that I am not allowed to be angry around them. (Brooke, FG1)*

*You just like go out, even if you just go out for a couple minutes and have a breather or something. (Abby, FG1)*

*They’re like sponges, aye [regarding infants’ behaviour being noticeably different when their parents are fighting compared to when their parents are happy]. (Abby, FG1)*

*Yeah, they’re like sponges. They absorb what they see and feel. (Erin, FG1)*

#### 3.2.3. Sources of Knowledge

There was evidence of sources of knowledge from each topic discussed, including the topics raised by the researcher, as well as those that the learners’ conversation naturally progressed onto. When it came to food safety during pregnancy, the midwife was the main source of information for all but one participant. Only one participant described social media as a source of information about food safety during pregnancy.

When discussing the topic of “hearing” specifically, the participants reported that they had not had any school-based science lessons on this topic (see Table 4).

There was evidence from the learner’s discussions that one source of knowledge was observing the behaviours of family members. This was true for many of the topics discussed.

*My aunty and uncle had this fat-as [big] argument, and my cousins were probably like five or six [years of age]. And them just sitting there in shock. I can tell…when their parents are happy, they’re always happy and excited and wanting to be around them, but as soon as their parents are like having a go at each other, they would just, “aye, oh what’s going on, I don’t want to be by them”…make them more stand-off….So if you feel like shit, don’t be around your kids. (Erin, FG1)*

Another source of knowledge was receiving direct advice from family members. Specifically, aunties, uncles, and mothers were mentioned by numerous participants while discussing a number of different topics.

*…I got told by my aunty…when she had my older cousin… she used to have like post-partum depression or whatever and she used to get like real mad, and when she was mad, she wouldn’t face him. She’d turn her back to him, so that he couldn’t feel her emotions. (Brooke, FG1)*

There was also evidence of learning via attentively observing the reactions of their infants.

*Like when baby had colic, and she was crying for too long, I’d just like, if I was getting frustrated it wouldn’t help her, it would just make her cry even more. Like, I wasn’t like frustrated like where other people could tell. But I don’t know, it was like she could feel my inside emotions. And so, I just put her down and went into the bathroom for a little bit. Had a breather and then went back out. And then she’d settle down. (Abby, FG1)*

*Do you think you just figured out by seeing her reactions? That that was a good thing to do? (Researcher, FG1)*

*Yep. (Abby, FG1)*

*Or did you see someone else do it… (Researcher, FG1)*

*Na, I just seen her reactions. (Abby, FG1)*

Aspects of knowledge learnt from family appeared to involve Māori culture or family culture.

*…Your family member giving you a growling… My mum used to say it [tinnitus] was my Nan [who had passed away], and she was... growling. (Abby, FG1)*

There was further evidence of Te Ao Māori knowledge that came from FG2; however, it was unprompted by the researcher and therefore has been included in this section of the analysis. The source of this knowledge was previous schools, where participants had learnt about Te Ao Māori. Darla was able to sing several songs (“pungawerewere” and “E toru nga mea”), which she described as remembering from Kohanga reo. When the topic of stories about Māui (pūrākau) was prompted by the researcher, all the participants knew at least one story. In addition to this, Abby was able to describe many pūrākau in detail, and this included the lesser-known tale about Māui Tikitiki-a-Taranga. She described her knowledge as having come from reading books at her previous school.

### 3.3. Teaching Tool Development

Four main themes were identified through RTA of FG2 and FG3 and influenced by the entire study:

1. Effectiveness of teaching tools;
2. Group learning helpful for all, despite there being individual learning needs;
3. “Busy” [families experiencing high strain];
4. Te Ao Māori (the Māori world).

#### 3.3.1. Effectiveness of Teaching Tools

FG3 revealed what the learners had understood and remembered from the previous week’s teaching session. There was evidence of the learners remembering and using some of the concepts or tools discussed the previous week. This included the concept of neuroplasticity:

*Filling them up with words…And why do we want to do that lots within the 0–2 period? (Researcher, FG3)*

*Because they have a big, what is it called, oh, because you are trying to mould their things, their connections! (Darla, FG3)*

*What is the property of slime, and play dough, that links to the concepts we were talking about last week? (Researcher, FG3)*

*It’s mouldable. (Darla, FG3)*

*Yes, that’s right. Can you remember any other terms? (Researcher)*

*Neuroplasticity! (Darla, FG3)*

*Yeah, excellent, well done. And when is the highest time of neuroplasticity for an infant? (Researcher, FG3)*

*When they are little… was it [up to] three [years old]?... or was it two? (Darla, FG3)*

Examples were noted of the participants acknowledging the importance of speaking to their infants and of implementing this over the week between FG2 and FG3. This included the importance of turn-taking conversations with pre-verbal infants and the use of engaging language, such as onomatopoeia.

*Like if she starts babbling, I’ll start talking back to her (Freya, FG3)*

*…The car seat one…so I was like “what sound does it make?” and then I go “click”. (Clara, FG3)*

It also included using songs with their infants during the week.

*I just sung those things to her, that you gave, those cards. [song cards, see Supplementary Material S2] (Abby, FG3)*

Darla reflected on the short-term nature of a behaviour change following the teaching session.

*I think straight after the talk I tried to talk more to Daniel. (Darla, FG3)*

*Cool. (Researcher, FG3)*

*But that was the only time I’ve tried. (Darla, FG3)*

*Haha, did you not try again after that? (Abby, FG3)*

*Well, just like actually trying, but otherwise it’s just been normal, like what I normally do. (Darla, FG3)*

Some concepts were not well remembered, which included the concept of the “handful of language”. This was a cue about how to fill an infant up with language, by having a 4 to 1 ratio of statements to questions (see Supplementary Material S2).

*Wait sorry, what? A hand? (Darla, FG3)*

*Yea, a handful of language, as a little cue. It’s a tip on how to interact with talking with your infant. (Researcher, FG3)*

*Oh, the um Deaf one? Oh no, wait. Oh, um the spider one? (Clara, FG3)*

*No. Oh, Pungawerewere? That was just… (Researcher, FG3)*

*Ok, what are we actually talking about? (Clara, FG3)*

*We talked about a helpful…tip about how to speak to your infants is to use four statements for every one question. (Researcher, FG3)*

*Aye? Na, I don’t remember that one. (Clara, FG3)*

*Neither, sorry. (Darla, FG3)*

*We had the little harakeke balls when we were talking about it last time. (Researcher, FG3)*

*Oh, I was playing with that [harakeke ball]. And looking at it. Weren’t we just talking about tapu? (Abby, FG3)*

There was evidence that EF can be a difficult topic to understand. It was explained in the FG2 teaching session during the talk about neuroplasticity and the purpose of language exposure in infancy.

*Do you remember what any of those [executive functions] were? (Researcher)*

*No. (Darla, FG3)*

*Laughter (all participants, FG3)*

*[with further prompting, Freya had volunteered “self-control”]*

*Can you think of any other higher level thinking things we talked about last time? (Researcher, FG3)*

*Silence (all, FG3)*

*Did we talk about that last time? (Clara, FG3)*

The conversation naturally flowed during FG2 to some reflection about whether there was a preference for learning about the importance of language in the 0–2-year-old via neuroscience and population associations or from drawing learnings from pūrākau.

*Oh, well, I liked them both. I liked like the scientific research kind of stuff behind this. But I liked the traditional one because, I don’t know, just probably because I’m a Māori. (Abby, FG2)*

*The part out of the story that really opened my eyes [made me stop and think/gave me understanding/reminder/realisation] was…that Māui heard his brothers’ names while he was in the womb. So… I knew like while I was pregnant, cause my midwives always told me “talk to your baby because they will recognise your voice…” but I didn’t really think much of it, ‘cause I thought, “oh, they won’t really remember it”. (Darla, FG2)*

Earlier in the session, the difference between children remembering experiences and infants’ brain architecture being moulded by experience was discussed, including the notion that although the latter does not lead to memories, it does have a lifelong impact.

Social media did not appear to be a major influence for the participants for the study topics. One participant indicated that she had interacted with one of the researcher’s prescribed social media sites (Abby). This may have been influenced by the longer time she had had to interact with the social media sites, as the researcher and Abby had chatted about the sites during the whakawhanaungatanga time. Alternatively, it may reflect a property that was unique to Abby as she was the only participant to mention social media as a source of knowledge for learning about diet during pregnancy in FG1.

An alternative option to practical descriptions of EF could be to avoid this concept altogether when explaining the purpose of hearing in the 0–2-year-old time. One participant explained in FG3 that she believed a good reason to support infants early-life language development was that this in turn enables them to express themselves, and this provides them with the confidence to communicate with their teachers and peers at school. Supporting her son to become confident was an important and motivating factor for her. It may be that confidence is easier to understand and more meaningful than EF as a purpose for supporting hearing for language in the 0–2-year-old time for AP learners.

#### 3.3.2. Group Learning Helpful for All

There was evidence of a variety of individual learning needs and abilities within the group. This became evident when ‘Participant B’ was away from the group while ‘Participant A’ answered a question about the purpose of providing an infant with exposure to language. This was during a recap of the previous week’s teaching session.

*…Because you are trying to mould their things, their connections! (Participant A, FG3)*

When Participant B returned:

*[Why might we] want to fill them up with lots of language? (Researcher, FG3)*

*Why? So, they can understand what you are talking about. [pause] How much do I have to give? (Participant B, FG3)*

There was further evidence later in the discussion that Participant B had not completely grasped the broader concepts about the purpose of providing lots of language to infants.

*[It had been Participant B’s turn to practice “a handful of language”. She had given four statements and was now thinking of a question.]*

*I don’t know. (Participant B, FG3)*

*“Do you like this?” (Participant A, FG3)*

*Oh, “do you like this?” (Participant B, FG3)*

*Yeah, yep. (Researcher, FG3)*

*Oh, or I’ll just ask him what the colour was. (Participant B, FG3)*

*Yep, you could do that. [said hesitantly] (Researcher, FG3)*

*To see if he would know. (Participant B, FG3)*

The last line by Participant B implies that the purpose of her question is to test the knowledge of the child. The purpose of the “handful of language” technique had been to emphasise the importance of language exposure and to reduce the main carer’s expectation of answers from the infant.

Despite the individual learning needs and speeds of the participants within the group, there was evidence that the group approach helped all the individuals. Natural peer support and peer teaching was observed between the participants. All the learners, on all attempts at a handful of language, became stuck trying to make four statements (four learners, three attempts each over two sessions). During a long pause, another learner would volunteer help by suggesting a word or phrase. Below is one occurrence of this:

*Um, oh, [exasperation] how do you explain this!? (Darla, FG3)*

*Colourful. (Abby, FG3)*

*Metallic. (Clara, FG3)*

*…Yeah, metallic colour… (Darla, FG3)*

During all three focus groups, there was evidence of the group helping each other find the correct words or concepts. They finished off each other’s sentences, offered encouraging words, or paraphrased one another’s comments.

*…So, they were really, I reckon kids are, just pick off by what they see. (Erin, FG1)*

*They’re like sponges, aye? (Abby, FG1)*

*Yea, they’re like sponges. They absorb what they see and feel. (Erin, FG1)*

#### 3.3.3. “Busy” [Families Experiencing High Strain]

There were many references by the AP learners to activities or responsibilities they had beyond schoolwork and childcare. These examples related to physical, emotional, cognitive, and social demands beyond what might be experienced by an adult parent or a parent not experiencing multiple strain factors, which are not due to being young but are commonly associated with adolescent parenthood.

One specific example was that AP learners often exhibited exhaustion due to sleep deprivation, likely from the many months of disrupted sleep from caring for their infants overnight. Grit was exhibited by many AP learners as they pushed themselves to progress through their academic schoolwork during the day.

Another example was that in FG sessions there were references to home life being busy. The word busy conveys two things. Firstly, that the learner is experiencing multiple strain factors, a disproportionate amount of burden compared to that experienced by an average parent. Secondly, it conveys that the learner has a determined attitude towards coping with her workload. “Busy” is not an objective measure, it is the AP learner’s perception and description. The amount of burden she copes with daily may be crippling for many adult parents.

*[Darla had described her infant opening a book and flipping the pages] That is what Ameilia does! But I’m guessing she wants me to read her a book when she does that. That’s why I go “oh not yet” or “mummy read you one tonight” because I’m usually busy like cleaning or something when she goes up to the books... (Abby, FG2)*

*So maybe like involving her with your cleaning can be another thing… (Researcher, FG2)*

*Oh no! Involving her with my cleaning just makes it messier. (Abby, FG2)*

*It does. It’s hard work. (Researcher, FG2)*

*It doesn’t even bloody help! [disgruntled, tired, overwhelmed, at her limit] (Abby, FG2)*

Further specific evidence from the FG session supporting this theme came from the tension that arose between the researcher and the participants regarding the topic of infants having screen time (watching a TV, laptop, cell phone, or tablet). The tension or pushback towards the researcher came from numerous participants. This tension continued despite the researcher framing screen time as being good for supporting parents to complete tasks or rest but was not enhancing for infant brain development.

*So, Miss Rachel [a kids’ TV programme], she makes them like repeat after her, and stuff like that…and she sings songs… (Abby, FG3)*

*She’s like “can you say mama”… (Clara, FG3)*

*…Then she starts making a song out of it. It is stuff that…you would want to do with your kid, but you don’t want to. (Abby, FG3)*

*Laughter (all, FG3)*

*So why wouldn’t you want to? (Researcher, FG3)*

*Na, you do want to, but say you were busy…then they go and watch that instead. (Abby, FG3)*

Abby uses this word “busy” for a second time. It encapsulates the relentless demands of cleaning and housework alongside the constant responsibilities of parenting. It reflects the emotional toll of being the sole adult managing these daily tasks within the home. Her tone conveyed frustration at being taught about ideal practices for engaging with her infant at home, while she feels she is already at full capacity with her workload, daily responsibilities, and mental exertion.

#### 3.3.4. Te Ao Māori

The theme “Te Ao Māori” (the Māori world) included participants showing enjoyment of ako (a Māori teaching pedagogy that is reciprocal), a high level of knowledge regarding Māori tikanga (Māori tradition cultural customs), and enjoyment of learning more about tikanga. It also included the fact that use of a pūrākau (traditional Māori story) was effective at maintaining the group’s attention. Further evidence came from remembering the discussion about tapu (sacredness discussed in the ako time) while struggling to remember the discussion about the language cue “a handful of language”.

During FG2, there was a spontaneous episode of the participants teaching the researcher. This appeared to be enjoyed by the participants. This was in line with the Māori teaching pedagogy of ako (reciprocal learning, where both the teacher and the student are learning and teaching).

*[Harakeke balls were handed out. It was explained that karakia had been performed prior to harvesting this harakeke]… She [researcher’s sister] got her Māori partner [to harvest the harakeke], ‘cause she was hapu [pregnant], so it’s too, it’s, I forget the word… (Researcher, FG2)*

*Tapu [sacred]. (Clara, FG2)*

*Yep…and you are not allowed to karanga onto the marae either when you are pregnant…(Abby, FG2)*

The conversation flowed into further explanations of Māori tikanga, with the “teacher” role of sharing knowledge swapping between the participants and all participants appearing to enjoy this shared teaching dynamic (ako), the conversation topic (tikanga Māori), and the fact that these were being encouraged rather than deterred by the researcher.

*…I didn’t know any of that! [happy and excited tone, following a peer participant’s explanation of tikanga] (Darla, FG2)*

The conversation included details of Māori tikanga around what is tapu while pregnant and certain aspects of tika (appropriate) conduct during a powhiri (a welcoming ceremony with high cultural importance) and while visiting an urupa (graveyard).

Further evidence for the enjoyment of learning about Māori tikanga came at the end of FG2. Immediately following the researcher stating that the session had finished, the participants’ conversation reverted back to the topic of Māori tikanga.

*See, I don’t even know any of these things! (Darla, FG2)*

*Don’t you! And you went to kohanga. (Abby, FG2)*

*‘Cause I went to kohanga, but then after kohanga I went to a kinda white school, and they didn’t really, like I didn’t know any of that. (Darla, FG2)*

*Why?… (Darla, FG2)*

*Because it is tapu!... (Carla, FG2)*

There was a clear interest in the pūrākau. Throughout the sessions there were regular interruptions. There was a noticeable contrast, however, during the pūrākau that the researcher read aloud during FG2. There appeared to be sustained attention during the reading of this story, as supported by the lack of interruptions on the audio recording. There was one interjection only, which was a comment on the story itself. The reading out of the pūrākau was 2 min and 44 s. The average time between interruptions that were unrelated to the topic of the focus group discussion during FG1 was 1 min and 30 s. The unrelated interruptions included phone beeps (12), verbal interjections about unrelated topics (prompted by a sound in the environment (2), prompted by a learner (4) or cross-talk/a side conversation about an unrelated topic (1).

There was evidence of pride in being Māori. One participant expresses that her infant prefers action songs that are in Te Reo Māori, inferring that she, as her infant’s main carer, has actively chosen to expose her infant to songs which are in Te Reo Māori rather than English.

*Oh, action songs. I don’t do that [head, shoulders knees and toes. Abby’s expression shows distain for this English action song]. I do Pipi Mā and Tākaro Tribe [action songs from TV shows that are in Te Reo Māori], ‘cause that’s what she likes. (Abby, FG2)*

A typical pōwhiri usually involves many people, but there is only one person who has the role of performing the karanga (one from each side: the guests *manuhiri* and the hosts *tangata whenua*). Performing the karanga requires skill, and the person chosen to perform this is well practised and coached by an expert. An individual with mana (well-respected) is chosen for the task, meaning they have had good behaviour and shown leadership aptitude. Performing the role is prestigious. These attributes were unspoken but appeared to be well understood by the participants in FG3. The excerpt below shows the pride that Clara felt about the fact she had performed a karanga at her previous school.

*[Abby had explained that she had not completed a real karanga but had practiced a karanga in a group at a Te Reo Māori class.]*

*Mine [karanga] was at intermediate. (Clara, FG2)*

*Really? You did the karanga? (Researcher, FG2)*

*Yep [said confidently and in a proud manner] …the one that goes [Clara then sang the first line of the karanga]. (Clara, FG2)*

## 4. Discussion

This study aimed to explore adolescent parents’ understanding of infant hearing and the sources informing their knowledge and to undertake teaching tool development. The population involved were not defined by any level of hearing but were a collection of families facing high strain. It is acknowledged internationally that EHDI programmes need to improve the way they interact with families facing high strain [12].

This study found that AP learners had some understanding of hearing and the importance of hearing for infants aged under two years. The results showed, however, that when describing the importance of multisensory interactions between the infant and their main carer, AP learners prioritised senses other than hearing in their descriptions. This, together with the evidence of limited discussion time spent on the topic, suggests that hearing may be an abstract or unfamiliar concept for AP learners. This may be generalisable to the families and communities surrounding AP learners, as they were identified as an influential source of their knowledge.

The identification of sources of knowledge was expanded to include all health topics discussed. The major sources of knowledge for this group of AP learners were midwives and family members. Learning from family members included both being given advice, as well as observing family members’ actions and the impact of these. Social media engagement as an information source was minimal, with only one out of nine AP learners reporting its use.

Teaching tool development involved the identification of four main themes: the effectiveness of teaching tools; group learning being helpful for all; “busy” [families under high strain]; and Te Ao Māori (the Māori world).

The teaching tools used in this study were effective for some topics, such as neuroplasticity. The study design included rapport building, the use of a safe space, multimodal teaching tools, and learner choice components. Multimodal teaching and learner preference-based approaches have well-documented benefits [45,46,47,48], however, they are not commonly employed by audiologists when sharing information with families [49]. Use of the AP learners’ safe space was welcomed by the TPU lead teacher and is in line with recommendations from the Growing Up in NZ study [6] and from overseas studies working with minority groups or those facing discrimination [50,51].

The findings from this study indicate that many participants adopted the techniques taught in FG2; however, one participant noted that this change was short-lived. Previous studies have shown the benefits of weekly sessions over a six-month period [52], and involving infants in the sessions has been associated with further benefits for families experiencing high strain [53]. Adopting these may have improved the longevity of the behaviour change.

Despite successfully communicating many concepts, the concept of EF remained abstract and less accessible within this context. Alternative methods for explaining EF to families from low socioeconomic backgrounds have been explored [33]; however, their effectiveness remains uncertain. Notably, an AP learner voluntarily articulated her motivation for supporting her infant’s language development: helping her son to be confident at school. This suggests that fostering future confidence may offer a more tangible framework than EF. Additionally, primary carers may differ in their motivations for supporting early language development. Co-design approaches, [37,51], where audiologists collaborate with main carers to set personalised goals, could achieve both teaching and elucidating main carers’ understanding, as well as aligning the motivation for early life language enrichment with their individual aspirations for their infants.

Group learning was helpful for all, as the peer interactions fostered knowledge sharing and self-expression among the AP learners. Mutual support and empathy were consistent aspects of the group dynamic, benefiting all participants. Previous research provides evidence for the benefits of group learning for parents [54], for families of children with long-term health conditions [55], and for Māori families [56], and it provides a unique form of support that professionals alone cannot offer [57]. Additionally, group learning with a professional facilitator has been recognised for improving efficiency in health education [58].

The theme “busy” encompassed both the strain the AP learners were under and also the resilience, perseverance, and grit that they demonstrated. For some, becoming a parent as an adolescent can mark a positive turning point in their life [59,60]. Adolescent parenting does not predict poor development outcomes for infants [61]; however, research shows that infants raised in environments with multiple strain factors have increased risk of poorer development [5,61], and that adolescent parenting is associated with additional strain factors, [13]. Providing safe spaces with wraparound support, such as a TPU, is crucial. For EHDI services, any infant identified with permanent hearing loss faces strain on their early brain development. Programmes require a multidisciplinary approach to explore the social context of each infant and identify families experiencing multiple sources of strain. Tailored solutions and a disproportionate investment of resources are necessary to support these families and work towards improved LTFU rates and equitable developmental outcomes for all families identified by EHDI.

A key finding of this study was the effectiveness of integrating Te Ao Māori principles and the positive response from AP learners to having access to traditional Māori teaching methods alongside Western scientific methods. The benefits of culturally appropriate teaching pedagogies align with Kaupapa Māori research principles, which advocate for culturally grounded health and educational interventions [36,62]. The advantages of Kaupapa Māori approaches have been demonstrated [63], and culturally grounded interventions for parents have been recognised as beneficial for Indigenous communities internationally [64]. The design of this study prioritised early rapport building, a key aspect of cultural safety in Māori health services [65,66].

The documentation of Indigenous worldviews in academic literature is expanding, with methodologies evolving to allow improved authenticity [64,67,68,69]. Acknowledging the influence of nuanced cultural differences in perceptions of infant development [68] highlights the limitations of earlier research aimed at supporting infants from high-strain families [70]. It is essential to consider parents’ cultural contexts as a fundamental aspect of the design of research and services.

While extensive research exists on supporting the wellbeing of AP learners and their infants, as well as infants from low socioeconomic families, an academic debate regarding appropriate approaches remains [13,71]. In NZ, this debate is added to by Māori academics with a high knowledge of a Māori world view [26]. Ware [26] questions whether prioritising academic qualifications to improve adolescent parents’ earning potential should take precedence over developing their parenting skills and time spent bonding with their infant and becoming responsive to their needs during their formative first 1000 days [67,72,73,74]. AP learners in this study described being “busy”, reflecting the extraordinary demands on their physical, emotional, and cognitive capacities. This context can create tensions between competing priorities, such as educational attainment and time for bonding.

### 4.1. Implications for Practice

This study’s findings suggest several practical implications for EHDI programmes for engaging with AP learners, which may be generalisable to many families experiencing high strain.

- Group learning activities should be integrated into EHDI programs;
- Teaching sessions should be frequent but short and tailored to the developmental stage of the infant;
- Where possible, interactions should take place in locations identified as safe spaces. Potential safe spaces include libraries, Plunket rooms, primary care settings, community centres, and marae (Māori community meetings spaces, with a high cultural value);
- Culturally appropriate teaching should be prioritised. This requires early investment in rapport building and understanding the caregiver’s cultural context, which may be shaped by ethnicity, family traditions, or chosen adult identities;
- A co-design approach to the EHDI journey will enable the main caregivers and families to express, in their own words, what motivates them to support their infant’s early language development.

### 4.2. Limitations and Future Research

Although the researcher supported screen time when used to facilitate parental rest or tasks, discussions surrounding screen time’s lack of positive impact on infant brain development led to some tension between the researcher and the participants. International research indicates that only a small proportion of families adhere to recommended screen-time limits for children [75]. The nuances of screen time, including its interaction with mealtimes [76], parenting styles [77], and adult co-viewing [78], are emerging areas of research. Future studies in this area may elucidate more helpful ways to communicate the science around screen time with families under high strain.

The study design restricted participation in later sessions, limiting the numbers involved. Future studies should explore a model of multiple short, open-access sessions. Additionally, while the findings are likely relevant to other families experiencing high strain, caution does need to be taken when applying the findings of this study to other populations. All the individuals in this population identified as Māori, and families facing high strain may be from different cultural backgrounds. Co-designed EHDI journeys with families would help identify both the family culture and individual motivations for supporting early language development.

## 5. Conclusions

This study highlights the importance of culturally grounded health interventions for families experiencing high strain. Group-based learning fostered peer support, hands-on multimodal teaching was effective, and culturally relevant materials and pedagogies enhanced engagement. Utilising an existing safe space for this community of AP learners facilitated rapport building and engagement. EHDI programs could better support infants from high-strain families by partnering with trusted community spaces, integrating with wraparound care networks, and delivering interventions in an engaging and culturally appropriate manner.

## Figures and Tables

**Table 1:** Study timeline.

Phase 1		Phase 2		
Whakawhanaungatanga/rapport building.		Focus group sessions.		
Introduction and short presentation.	Weekly, half-day, working alongside and being available for health questions for 4–6 weeks.	FG1 Ascertaining the views of AP learners about the role of hearing in infancy.	FG2 Using teaching tools to explain the purpose of hearing in infancy.	FG3 Evaluation and critique of the teaching tools.
Whole class.	Whole class.	Study participants only.	Study participants only	Only study participants who had been present at FG2.

**Table 2:** Participant pseudonyms, the focus groups they attended, and their infant’s pseudonym. The infants did not attend the focus groups.

Participant Number	Participant Pseudonym	Infant Pseudonym	Focus Group Sessions Attended
1	Abby	Amelia	1, 2, 3
2	Brooke	Blake	1
3	Clara	Carter	2, 3
4	Darla	Daniel	2, 3
5	Erin	Ella	1
6	Freya	Fox	1, 2, 3
7	Georgia	George	1
8	Hayley	Harper	1
9	Indie	Isla	1

**Table 3:** FG1 transcript content that related to the topic of infant hearing.

Category	Row #	Description of What Participants Discussed. (Researchers’ Words)	Transcript Excerpts. (Participants’ Words)
Sounds infants hear	1	There was a discussion about what infants typically hear.	Hayley: [They hear] their mum’s voice. Erin: white noise [can be used to help infants sleep].
	2	There was acknowledgement that infants can hear in utero.	Abby: [They can hear their mum’s] heart beats
	3	Infants can hear in utero, from 20 weeks’ gestation.	Researcher: Does anyone know what number of weeks in utero? Abby: Is it like 20, something? Researcher: Yeah, it’s 20. Abby: Oh, 20 exactly? Oh, shit!
	4	For some APs, music was routinely heard by their infants.	[The prior discussion was about sounds or phrases that are heard routinely.] Darla: A song when we are cleaning up. Researcher: When you are cleaning up? Darla: Yep, then I put the music on.
	5	AP learners thought that infants’ hearing may be more sensitive than adults’ hearing. This occurred after discussing that generally infants are quite sensitive to many things due to everything being new to them.	Erin: I think babies can hear volumes like, we think it’s normal, but to them it’s like… real high intensity.
Hearing checks	6	For two participants, the infant hearing screening machine touching the soft spot made them feel uneasy.	Brooke: I was just scared of the big machine. Researcher: Why was that? What was scary about the machine? Brooke: I don’t know, it was just where it went on his head… I don’t know Erin: Mmm [agreement]. Erin: And the head thing, actually, I didn’t like that it was going over her soft spot. Researcher: Ok. Which did you find worse, the heel prick or the hearing screen? Erin: Heel prick, definitely. Researcher: Ok Erin: For the hearing thing [check], she was asleep so… it was just that… it bothered me.
	7	For most participants, the hearing screen took place at a convenient location and was an unremarkable memory.	India: It [her own infant’s hearing screen] went good. Georgia: It went good. Researcher: Was it in the hospital or the birthing centre? Georgia: Hospital. Freya: Um, yeah it [her own infant’s hearing screen] was all good. Hayley: Was that with the gel on the…? [Hand actions to indicate head.] Researcher: Yep Hayley: At [local birthing centre]. Researcher: At [local birthing centre], cool. It makes it easy if they come see you there. Hayley: Yeah. Abby: Yeah, Amelia’s one was at the hospital, and she was 10 days, ‘cause we were getting discharged from NICU, and she failed it. …she just wouldn’t stay still. She was crying and stuff. Unsettled. So we went to the, um….I think we went to… [local birthing centre] as well [for the second attempt at the hearing screen]. …then she passed that one and everything was all good. They said she didn’t fail the first one, it was just because she was moving. They didn’t get to do a reading properly. Clara: I was at the hospital too [her own infant’s hearing screen]. Researcher: Before you were discharged? Clara: Yep.
Infant development	8	In response to the prompt ‘first 1000 days’, they thought that this was referring to the first three years of life being crucial for development.	Brooke: Isn’t that the first three years are crucial?
	9	AP learners showed knowledge of the importance of the bidirectional nature of main carer and infant interactions, and that this is more engaging for an infant compared with a sensory experience that does not involve the main carer, such as listening to music from a speaker.	Erin: [previous discussion about music, and the conversation topic changed to singing] I reckon kids interact more when you interact with them. Doing whatever it may be.
	10	AP learners showed knowledge of the importance of being expressive when interacting with infants.	Researcher: Have you guys been taught some things about the ways things impact babies over their first two years of life? Hayley: Yeah, like showing, like expressing the way you feel. Researcher: Yeah. Like being extra expressive with it [your communication]? Hayley: Yeah. Researcher: To help communicate things to them? Hayley: Yep
	11	AP learners showed awareness of the ways that pre-verbal infants can communicate using non-verbal responses. There was also awareness that infants were able to comprehend familiarity, despite not having the brain development to support episodic memory.	Researcher: Do you think they remember what they have heard? Abby: Yep Researcher: Yep. And how do they show that? Abby: Because they are familiar with it, when they hear it again. Researcher: Yep… How do they show familiarity do you think? …Can you think of any examples? Abby: Like they look for it? Like they look outside. Clara: They look to the side.
	12	Brooke describes the Lombard effect, which she has observed in the behaviour of her son. This is a concept that is specifically about hearing and sound. This sharing occurs at the very end of FG1, perhaps showing that there was discussion and thinking about many things that were close to or interrelated with hearing, and perhaps for this AP learner, she was able to progress in her thinking over the session towards identifying and sharing a story that was specifically related to the hearing sense. As well as the Lombard effect, Brooke’s final thoughts, shared quickly at the end of the session, included her infant’s imitation of speech and voice tone, as well as the rhythm and pitch of music. The achievement of these milestones in expressive language indicates that her infant has had good hearing and a good language environment over their first 12 months of life. It also illustrates that Brooke has an awareness of these early and subtle infant development milestones, suggesting she is well bonded with her infant and responsive to his subtle expressions.	Brooke: Oh, um, I noticed that when we were in, like, a loud place and there were lots of people talking or there is loud music, he gets really loud. Researcher: Oh, he gets loud with his own voice? Brooke: Yeah. Abby: Does he like match the volume in the room or something? Brooke: Yeah. Researcher: Cool, that’s good. Brooke: Yea. And he likes mimicking people and their voice, and he likes to sing songs, like, he doesn’t sing it proper but…you know…[giggle. The giggle suggested she was unsure about whether she should be articulating this story to the group and the researcher]. Researcher: Can you tell the tune? Brooke: Yeah. Researcher: Cool. How old is he? Brooke: One. Researcher: One, oh yep. Oh, that’s very good. Cool.
	13	Music was raised by a participant as something that infants hear often, and she then expanded on this as she linked hearing music to the infant’s emotions and described it as something that was supportive of infant development.	Erin: Music is a good tool for like happiness with babies I reckon.
	14	Infants can detect some emotions of their main carers, and this has the potential to influence their developmental trajectory. The discussion was about a multisensory experience for infants, of which the hearing sense and the voice of the main carer are a significant portion of the interaction. The mother’s voice had been previously identified in FG1 as something infants hear often.	Brooke: I got told that I am not allowed to be angry around them… Erin: They read, off like, you know [they are able to detect the emotions of their main carers when the parent conveys this in an expressive manner. When these interactions are harsh, they have the potential to negatively impact the developing infant. This is a multisensory experience for the infant, which the parents voice (infant’s hearing) is part of] … Brooke: …’Cause I got told, um, by my aunty when she had my older cousin, um, she got real mad, oh, ‘cause she used to have like postpartum depression or whatever and she used to get like real mad. And when she was mad, she wouldn’t face him, she’d turn her back to him, so that he couldn’t feel her emotions.
	15	In this fast and flowing conversation, Abby supports Erin by providing an analogy for the point she is trying to make using a story about her family. Abby’s input was appreciated by Erin, as it conveyed more succinctly what she was trying to express. Erin then expanded on this analogy using the terms “see and feel”. There is an absence of the word “hear”. It is noteworthy that the hearing sense may not be as prominent or front-of-mind as the other senses when AP learners think about infant development.	Erin: Um…yeah I’ve…..My aunty and uncle had this fat as argument and my cousins, and I was six [years old] and [I remember] just sitting there in shock. I can tell if, you know like, when their parents are happy, they’re always happy and excited and wanting to be around them, but as soon as their parents are like having a go at each other, they would just aye, oh I don’t want to be by them. And then they would make them more standoff. So they were really… I reckon kids are just pick off by what they see. Researcher: Yep Abby: They’re like sponges, aye. Erin: Yeah, they’re like sponges, they absorb what they see and feel [infant hearing is also part of how these children would have detected the emotions of their parents in the described story, but the word hear was not stated by Erin].

**Table 4:** All prompts within FG1 that resulted in an absence of discussion.

Question or Prompt from GC	Absence of Discussion
Have you had any school science lessons about hearing?	No (all participants, FG1)
Have you ever heard of the “first 1000 days” concept or phrase?	No (many participants, FG1) [silence] Isn’t that the first three years are crucial? (Brooke, FG1) [No further expansion or discussion; the group conversation quickly moved onto the surprise that the first 1000 days started at conception]
Have you ever heard of the “first 1000 days” concept or phrase? [asked to the whole group a second time later in the discussion]	[silence] I don’t know (Clara, FG1)

## Data Availability

The data sets generated and analysed during this study are transcribed focus groups. They are not available for sharing due to maintaining the anonymity of the participants and the conditions of the ethical approval.

## Supplementary Materials

The following supporting information can be downloaded at: https://www.mdpi.com/article/10.3390/children12050629/s1, Supplementary Materials S1 and S2.

## Abbreviations

ABR	Auditory Brainstem Response
AP	Adolescent Parent
EF	Executive Function
EHDI	Early Hearing Detection and Intervention
FG	Focus Group
LTFU	Loss to Follow-Up
NZ	New Zealand
TPU	Teen Parent Unit
