# Supplementary material for: Partnering with Young Parents to Improve Early Hearing Detection and Intervention Programmes
Source: Children (Basel). 2025 May 13;12(5):629. doi: 10.3390/children12050629 (PMC12109589; doi:10.3390/children12050629)
Supplement: Supplementary file 1 [file children-12-00629-s001.zip › children-3641638-supplementary.pdf]

Supplementary Material $S1$

Research Flyer

Hearing for pēpi

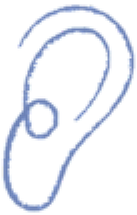

Research Flyer

Kia ora, I am a researcher and an audiologist, or kaimahi rongonga (hearing health worker). I'm completing my Ph.D. on hearing for 0-2 year olds in Aotearoa New Zealand.

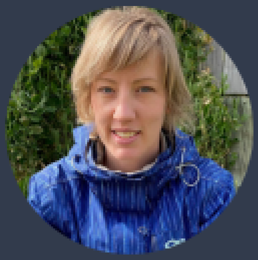

Gem Choi

Ko Tararua me Ruahine ngā maunga
Ko Manawatū te awa
Ko te Dominion Monarch te waka
Nō Ukaraina, Rūhia, Ingarangi ōku tupuna
I tipu ake au i Te Papaioea
E noho ana ahau ki Kirikiriroa
Ko Peter Choi tāku hoa rangatira
Tokorua ā māua tama
Ko Gem Choi tōku ingoa
Ka tukuna ngā mihi maioha ki a koutou

Contact details

Genevieve (Gem) Choi
PhD candidate, Dept of Audiology
The University of Auckland
gem.choi@auckland.ac.nz
021 121 5959

Research Supervisors

Dr Andrew Wood
Ear Nose and Throat Specialist and Senior Lecturer in Surgery

Prof. Suzanne Purdy
Head of School of Psychology

Assoc. Prof. Holly Teagle
Clinical Director for The Hearing House and Associate Professor in Audiology

Pātai / Questions?

If you have any questions, please contact Gem or your He Puaawai Kaiako

Whakawhanaungatanga (getting to know each other)

To help Gem and the get to know the learners and Kaiako at He Puaawai and vice versa there will be:

1. korero me te kai.

Gem will bring a lunch to share, do an introduction on her work, and chat with kaiako and learners over lunch.

2. weekly half-day sessions for 4-6 weeks

During the weekly half day sessions Gem will be working on her laptop in the classroom. During this time, learners are welcome to ask her questions. Here are some of the things you could ask her about:

- Hearing tests. If you would like your child to have a hearing test onsite at He Puaawai please ask Gem. Gem would then arrange to have the equipment with her on a future visit. Gem would talk about the test and the results with you and give you a written report. A copy of the report would also be sent to the General Practitioner or Well Child health provider.

- Hearing health. If you have a hearing or ear health related appointment for your child, you are welcome to talk to Gem about this. It may be that Gem could attend this with you.

- General health. You can ask Gem questions about health. She is not a doctor or a nurse, but she can help find relevant information online, and talk with you about a sensible next step for seeking health advice.

Following the 4-6 weeks of whakawhanaungatanga Gem will be doing a research study. The research study will involve focus group sessions with a few people from the He Puaawai class.

Participation in the research

If your first child is under 2 years of age, please consider taking part in the research study. Your kaiako will give you a Research Study Information Sheet which has the details of the study. The purpose of the study is to improve how we communicate about hearing and hearing healthcare for pēpi. There are Prezzy cards for participation.

THE UNIVERSITY OF AUCKLAND
Te Whare Wānanga o Tāmaki Makaurau
NEW ZEALAND

Participant Information Sheet

Hearing for pēpi

Research Study
Information Sheet

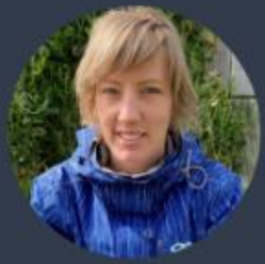

Gem Choi

Ko Tararua me Ruahine ngā maunga
Ko Manawatū te awa
Ko te Dominion Monarch te waka
Nō Ukaraina, Rūhia, Ingarangi ōku tupuna
I tipu ake au i Te Papaioea
E noho ana ahau ki Kirikiriroa
Ko Peter Choi tāku hoa rangatira
Tokorua ā māua tama
Ko Gem Choi tōku ingoa
Ka tukuna ngā mihi maioha ki a koutou

Contact details

Genevieve (Gem) Choi
PhD candidate, Dept of Audiology
The University of Auckland
gem.choi@auckland.ac.nz
021 121 5959

Research Supervisors

Dr Andrew Wood
Ear Nose and Throat Specialist and Senior Lecturer in Surgery

Prof. Suzanne Purdy
Head of School of Psychology

Assoc. Prof. Holly Teagle
Clinical Director for The Hearing House and Associate Professor in Audiology

THE UNIVERSITY OF AUCKLAND
Te Whare Wānanga o Tāmaki Makaurau
NEW ZEALAND

Kia ora, I would like to invite you to take part in the research I am doing as part of my PhD in Audiology. I am an Audiologist, or Kaimahi Rongonga (hearing health worker) and a researcher.

Key Information

Why are we doing this research?

We want to improve how we talk about hearing and hearing healthcare for pēpi. If parents understand the topic of hearing for young infants, they can then make educated choices about hearing checks and language options.

What will taking part involve?

Taking part will involve three focus group sessions. Each session will be 45 min and take place onsite at He Puaawai during a lunch break. Kai will be provided. As an expression of gratitude and to compensate you for your time, we will provide you with a $25 prezzy card at each session you attend.

- Session 1: Your view of hearing.
- Session 2: Gem's teaching tools.
- Session 3: Your feedback and critique of Gem's teaching tools.

Each session with be audio recorded. Gem will ask a series of questions, prompts and pictures which she will present over the 45 minutes to help facilitate the group discussions on the topic. The recording will be analysed for themes or ideas. The themes of session 1 will guide session 2. The themes of session 3 will help improve the teaching tools for future use and research. The themes of all the sessions will contribute to writing an article, and the reserach team aim to publish this in a scientific journal.

Checking the information.
The written down themes from session 1 and 3 will be made available to you after the session. You are welcome to add or remove information. You do not have to read or alter the themes if you do not wish to.

Seeing the results of the research.
This will be available a year after the sessions. Gem can email the results or can come and present the results at He Puaawai.

Pātai / Questions?

If you have any questions, please feel free to contact any of the below options:

- Gem
- A He Puaawai Kaiako
- Audrey D'Souza on:
  Group Services Co-ordinator
  The University of Auckland
  School of Population Health
  a.dsouza@auckland.ac.nz
  09 373 7599 ext.85536
- An independent health and disability advocate on:
  0800 555 050
  advocacy@advocacy.org.nz

Māori cultural support

We encourage you to discuss this research with your whānau and friends and to seek any cultural support you need from your whānau. Alternatively you can contact the administrator for He Kamaka Waiora (Māori Health Team): 09 4868324 ext 2324

Ethical concerns

You can also contact the health and disability ethics committee (HDEC) that approved this study on:

Email: hdecs@health.govt.nz
Phone: 0800 400 569
(Ministry of Health general enquiries)

Ethics Reference

Approved by Health and Disability Ethics Committees on 8 December 2023.
Reference: 2023 FULL 18720

THE UNIVERSITY OF AUCKLAND
Te Whare Wānanga o Tāmaki Makaurau
NEW ZEALAND

What are the risks and benefits of being involved?

- You will need to give up your lunch break to do each of the three focus group sessions. Kai will be provided.
- You may learn something new about hearing.
- You will be contributing towards improving hearing care services in Aotearoa New Zealand.
- You will receive a $25 prezzy card for each session you attend.

What are my rights if I take part?

Taking part in the research is completely voluntary and you will not be disadvantaged if you decide not to participate. If you choose to participate, you can change your mind at any time, including after giving consent, without providing a reason, and without any negative consequences.

Confidentiality

All personal information that may make you identifiable will be removed during analysis. Only the research team will have access to the information you share. The research will go towards published scientific articles, and these will not have any information that would allow someone to identify you personally. The research will describe the results as the views of adolescent, first-time parents on the topic of hearing.

What happens after the research ends?

The consent forms, which contain identifiable information, will be stored for ten years in a safe and locked storage space. The electronic information will be stored securely in a University of Auckland password-locked computer. Only the research team will have access to the consent forms and electronic information. After ten years, from when ethics approval was provided, the data and consent forms will be permanently deleted.

Who pays for the research?

Funding for this research has been obtained from Braemar Charitable Trust, Linsell Richards Education fund and Lottery Health Research Board. Being involved will not cost you anything.

Injury during the research study

If you were injured while taking part in this study, you would be eligible to apply for compensation from ACC just as you would be if you were injured in an accident at work or at home. This does not mean that your claim will automatically be accepted. You will have to lodge a claim with ACC, which may take some time to assess.

Supplementary Material S2

Focus Group 1 Visual prompts

An A4 piece of paper with the following topics typed in large font were placed in the middle of the group with each change in researcher prompted topic. These were:

1. Hearing
2. Infant hearing
3. First 1000 days
4. Hearing checks

Focus Group 2 Teaching tools

Teaching tools were developed based on the above analysis of the learner’s current knowledge and their typical sources of knowledge.

Concept 1: Neuroplasticity.

Activity: Guided play dough making

Participants were guided through the following steps by GC. A mixture of play dough, printed pictures and videos. Discussion was intermixed throughout.

Each participant was given a portion of play dough. They were guided through cutting this into thirds and joining two sections together. There was a concurrent discussion between GC and participants about the brain doubling in size between age 0 and age 2 years. Following this, a paper printout below was added to the work surface.	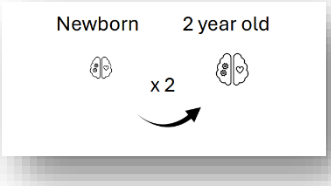
One participant's set of play dough showing the size growth between birth and 2 years, was left to one side. Each participant was then guided to each make a simplistic neuron from their play dough. A more detailed example of a neuron was shown, with a paper printout being added to the work surface.	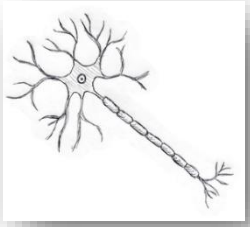

Each participant's simplistic neuron was added to a group structure which was central on the work surface. A visual representation of touch, sight, and hearing was added, along with a brain, in the arrangement shown. Plasticity was shown by manipulating the paly dough neurons and explaining what happens when the neuronal pathways are stimulated and when they are not stimulated in early life. The neurons grew many new dendrites. When a pathway was stimulated, the effect of strengthening the connection between the neurons was demonstrated with the play dough. When there was no stimulation, the play dough dendrites were removed.

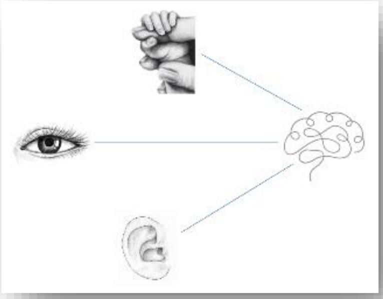

Examples of an extreme absence of stimulation were described, and whole neurons were removed. In these examples, other senses became connected to that pathway leading to the brain. This represented, for example, that in the absence of hearing, the auditory cortex becomes stimulated by visual stimuli.

Neuroplasticity was described as the brain being moudable. The generation of many new dendrites and the removal of the connections which were not being stimulated was described and shown using the play dough. It was explained that the brain is highly mouldable while under 2 years of age and becomes less so during the third year of life. Beyond this age, the brain is still mouldable but much less so than an infant's brain.

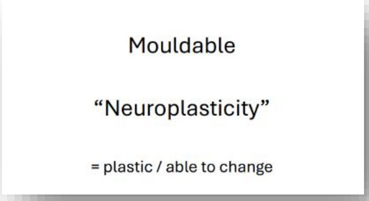

It was explained that although the brain doubles in size between age 0 and age 2 years, the number of neuronal cells does not change, and it is the number of connections that change. A printout of this picture was added to the work surface. It was explained that this allows the growing infant to be responsive to the environment. An example given was that the accent/tonal aspects of the parents' language are detectable in an infant's babble as early as six months of age. Also, some adults who are new to learning a language as an adult have difficulty in detecting sound differences that have no meaning in their native language due to the adult brain being much less plastic than an infant's brain.

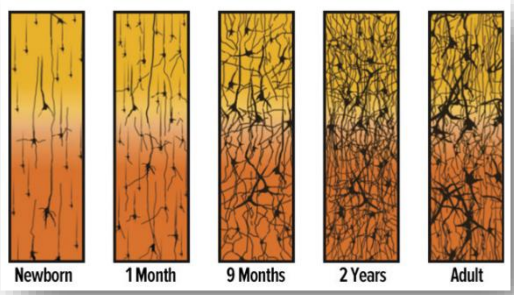

A video was not shown at this point. If repeating the teaching session, this example of pre-speech infant babble displaying the Liverpool accent of the parent and parent's friend would be shown: https://youtu.be/hkI8PmD8gHA

Good practices to support infant brain development (and strengthen neuronal connections) were explained. This was illustrated with reference to the central structure we had all created on the work surface using play dough. Touch and showing love through hugs and baby massage were mentioned (the participants had had a session with baby massage in the previous weeks). Other examples focused on the sense of hearing and the creation of a language-rich environment. These included providing a high volume of words, having pretend

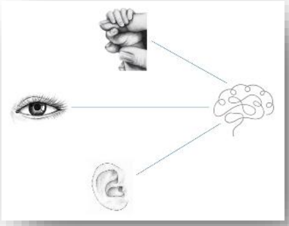

conversations with infants even before they have any of their own words, and exposing them to a large range of different words (a breadth of vocabulary).	
Having conversations with infants before they have clear single words was demonstrated using a video on a tablet. This was the video: [Kingston's conversation with Me over next season]	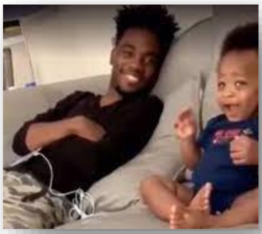
After the video was shown, a printed picture was added to the work surface to provide a visual representation of turn-taking conversations and being responsive to infants.	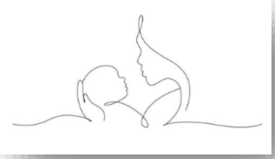
A visual representation of providing infants with many words was achieved by adding this printed square of paper to the work surface.	Moe moe pēpi
A visual representation of providing infants with repetition of words was achieved by adding this printed square to the work surface.	**Milk**!<br>**Milk** please, mummy.<br>I want my **milk**.
A visual representation of providing infants with a breadth of vocabulary was achieved by adding this printed square to the work surface. Later, a participant used the word 'crescent' when describing the shape of a banana. This was linked to this visual prompt as a great example of exposing her infant to a breadth of vocabulary.	Exciting!
It was explained that by providing a high volume of words, a breadth of vocabulary, and repetition, parents can support their infant's brain in forming strong connections for language. It was further explained that strong connections for language help the brain progress to its next stage of development: complex thinking or 'executive function.' Practical examples of different types of executive function were discussed.	Complex thinking<br>"Executive function"<br>e.g. working memory, flexible thinking, and self-control.<br>Trouble with executive function can make it hard to focus, follow instructions and control emotions.

Concept 2: Methods to Enrich an Infant's Home Language Environment

Activity 1: Songs

One video of a song was watched on the tablet (*Pungawerewere*), the group sung two songs together (*E Toru Ngā Mea, Kina Kina)* and the group *(Teddy Jumps*).

Printouts of each song, which included the QR code for the accompanying YouTube video, were given to each participant to use in the session and then take home. White tack was provided to allow participants to put these on a wall at home if they wished. There was dislike of the *Teddy Jumps* song and participants chose an alternative song which has meaning to them, and was appropriate for a young infant.

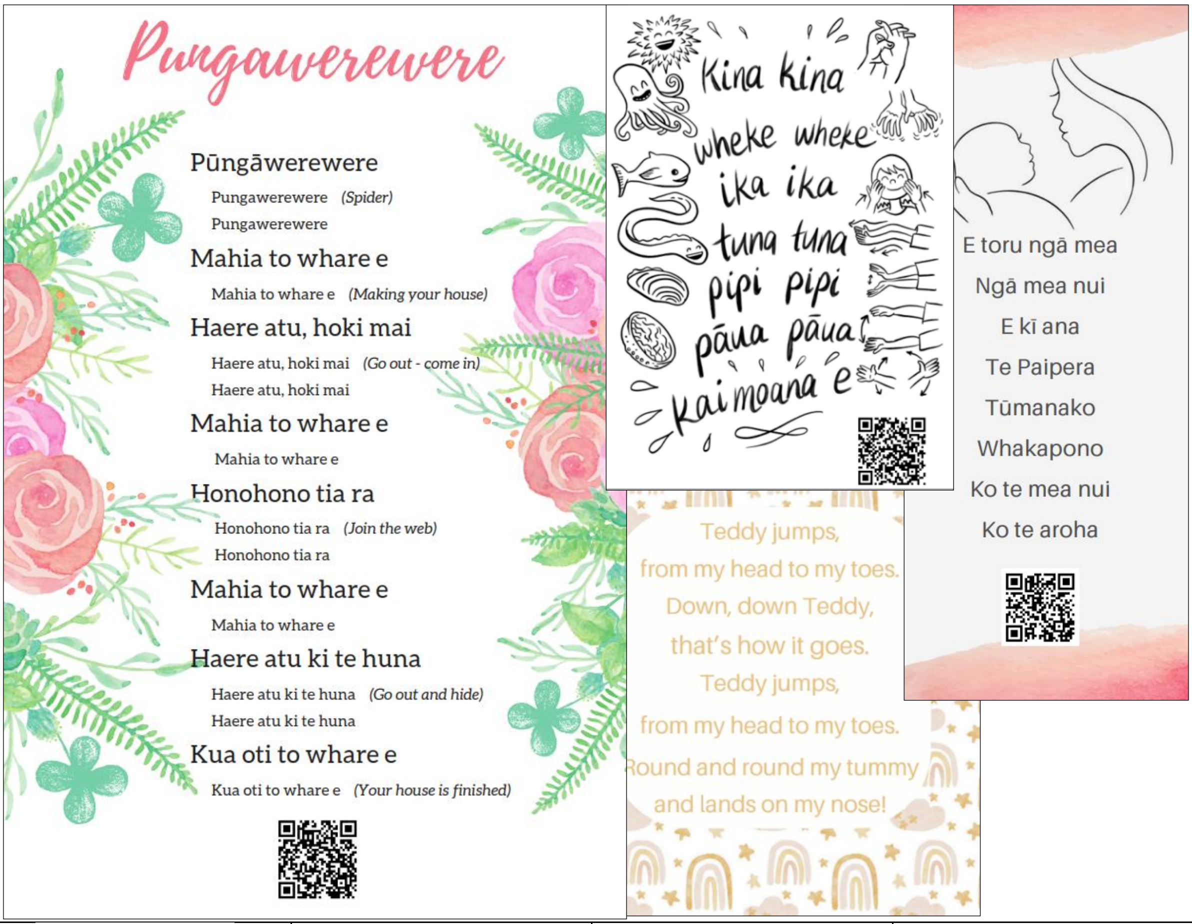

Activity 2: A Handful of Language

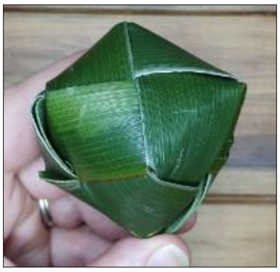

Activity: Each learner was given a ball made of flax. The researcher provided an example of four statements and one question, using a cow as the subject and her own hand as the reference. The participants were then invited to create their own "handful of language" using the flax ball as the topic, if they wished. They were encouraged to take the flax balls home at the end of the session.

Activity 3: Social Media

Social media sites (Facebook, Instagram, YouTube) were discussed. A paper handout was provided with QR codes linking to three recommended sites. Learners were encouraged to like or follow these sites at home or on their phones during the session. The sites included: Zazi Plays, Tākai, Tupuna Parenting, and Talking Matters.

It was explained that following reputable social media sites or personalities can lead to receiving easy reminders of positive techniques for interacting with infants during day-to-day social media engagement.

Concept 3: Infants' Ability To Hear Starts While They Are In Utero

Activity: Traditional Māori Stories (Pūrākau)

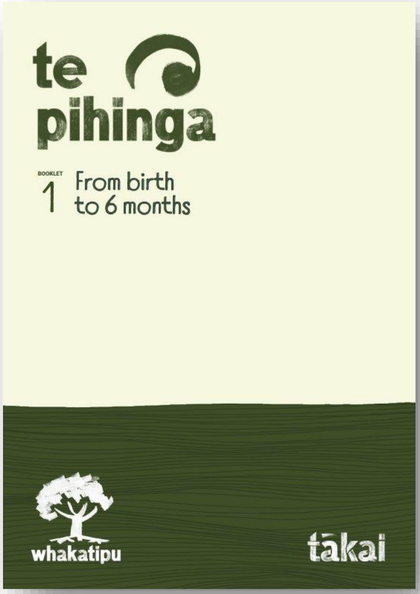

A traditional Māori story (pūrākau) was read aloud. These stories can be used to teach important life lessons that hold cultural significance for Māori and are commonly used to transfer knowledge between generations. The story was read from a booklet developed by Tākai. Five different booklets were created, covering ages from pregnancy to five years old. The participants were invited to take home a set of these booklets if they wished.

The specific story read aloud was *Māui Tikitiki-a-Taranga*. One of the lessons that can be drawn from this story include the fact that infants are able to hear while they are still in utero.
